# Comparative genome analysis and the genome-shaping role of long terminal repeat retrotransposons in the evolutionary divergence of fungal pathogens *Blastomyces dermatitidis* and *Blastomyces gilchristii*

**DOI:** 10.1093/g3journal/jkae194

**Published:** 2024-08-20

**Authors:** Lisa R McTaggart, Thomas W A Braukmann, Julianne V Kus

**Affiliations:** Microbiology and Laboratory Services, Public Health Ontario, 661 University Avenue, Toronto, ON M5G 1M1, Canada; Microbiology and Laboratory Services, Public Health Ontario, 661 University Avenue, Toronto, ON M5G 1M1, Canada; Department of Laboratory Medicine and Pathobiology, University of Toronto, 1 King's College Circle, Toronto, ON M5S 1A8, Canada; Microbiology and Laboratory Services, Public Health Ontario, 661 University Avenue, Toronto, ON M5G 1M1, Canada; Department of Laboratory Medicine and Pathobiology, University of Toronto, 1 King's College Circle, Toronto, ON M5S 1A8, Canada

**Keywords:** *Blastomyces dermatitidis*, *Blastomyces gilchristii*, whole genome sequencing, MycoSNP, species tree, long terminal repeat retrotransposons, genome contraction, fungal evolution, speciation

## Abstract

*Blastomyces dermatitidis* and *Blastomyces gilchristii* are cryptic species of fungi that cause blastomycosis, an often severe disease involving pulmonary infection capable of systemic dissemination. While these species appear morphologically identical, differences exist in the genetic makeup, geographical range, and possibly the clinical presentation of infection. Here, we show genetic divergence between the cryptic species through both a *Blastomyces* species tree constructed from orthologous protein sequences and whole genome single-nucleotide variant phylogenomic analysis. Following linked-read sequencing and de novo genome assembly, we characterized and compared the genomes of 3 *B. dermatitidis* and 3 *B. gilchristii* isolates. The *B. gilchristii* genomes (73.25–75.4 Mb) were ∼8 Mb larger than the *B. dermatitidis* genomes (64.88–66.61 Mb). Average nucleotide identity was lower between genomes of different species than genomes of the same species, yet functional classification of genes suggested similar proteomes. The most striking difference involved long terminal repeat retrotransposons. Although the same retrotransposon elements were detected in the genomes, the quantity of elements differed between the 2 species. Gypsy retrotransposon content was significantly higher in *B. gilchristii* (38.04–39.26 Mb) than in *B. dermatitidis* (30.85–32.40 Mb), accounting for the majority of genome size difference between species. Age estimation and phylogenetic analysis of the reverse transcriptase domains suggested that these retrotransposons are relatively ancient, with genome insertion predating the speciation of *B. dermatitidis* and *B. gilchristii*. We postulate that different trajectories of genome contraction led to genetic incompatibility, reproductive isolation, and speciation, highlighting the role of transposable elements in fungal evolution.

## Introduction

Blastomycosis can be a serious fungal infection. It often presents as a pulmonary illness, but systemic disease can also occur after hematogenous dissemination. While direct cutaneous inoculation is a potential route of infection, blastomycosis is most often acquired through inhalation of spores from the environment. Once inhaled, spores undergo a thermally dependent morphological transition to yeast cells which cause disease (Pullen *et al.* 2022). It is estimated that approximately 50% of *Blastomyces* infections are asymptomatic and self-resolving. However, serious pulmonary infections causing acute respiratory distress syndrome do occur (Pullen *et al.* 2022). Without treatment the infection is able to disseminate to other body sites including skin, bone, genitourinary tract, and central nervous system (Pullen *et al.* 2022). Depending on patient demographics, mortality rates range from 4–22% (Khuu *et al.* 2014).

Historically, blastomycosis infections were ascribed to *Blastomyces dermatitidis*, which is endemic to the eastern half of North America, particularly in areas along the Ohio and Mississippi River Valleys, and the Great Lakes (Ashraf *et al.* 2020). Cases of African and Middle Eastern blastomycosis are attributed to newly described species *Blastomyces percursus* and *Blastomyces emzantsi* (Schwartz *et al.* 2021), while *Blastomyces helicus* has been implicated in a few blastomycosis cases in western Canada and the United States (Schwartz *et al.* 2019). Several studies demarcated genetic groups among clinical isolates of North American blastomycosis cases (Meece *et al.* 2010, 2011; Brown *et al.* 2013; McTaggart *et al.* 2016), which led to the description of 2 cryptic phylogenetic species, *Blastomyces gilchristii* and *B. dermatitidis* (Brown *et al.* 2013). Incidence rates of blastomycosis caused by *B. dermatitidis* and *B. gilchristii* suggest a broad and expanding geographic range of endemicity with localized regions of hyperendemicity, raising increased public health concern in North America regarding these fungi (Seitz *et al.* 2014; Brown *et al.* 2018; Ashraf *et al.* 2020; Mazi *et al.* 2023). *Blastomyces* spp. exist in the environment as a filamentous mold which produces spores but little is known about their specific environmental niche(s) owing to the fact that attempts to isolate these fungi from environmental sources have been largely unsuccessful (Pfister *et al.* 2011). Recent PCR-based soil surveillance has shown promise as an environmental monitoring technique (Jackson *et al.* 2021).

While morphologically indistinguishable, several differences exist between the cryptic species *B. dermatitidis* and *B. gilchristii*. Genetic differences and reproductive isolation have been described based on multilocus sequence typing (Brown *et al.* 2013) and multilocus microsatellite typing (Meece *et al.* 2011; McTaggart *et al.* 2016). The geographic ranges inhabited by the 2 species also differ. Based on genetic analysis of clinical isolates, *B. dermatitidis* are recovered from cases throughout the North American range, while *B. gilchristii* appears to be restricted to several Canadian provinces and a few north-eastern US states (McTaggart *et al.* 2016). While both are capable of causing blastomycosis (Dalcin *et al.* 2016; Frost *et al.* 2017; Laux *et al.* 2020; Kaplan *et al.* 2021), data suggests that variation in clinical disease presentation may exist. In studies that differentiate the 2 species, *B. dermatitidis* has been observed to be more likely associated with disseminated disease while *B. gilchristii* appears to primarily cause pulmonary-exclusive infections (Meece *et al.* 2013; Frost *et al.* 2016; Fritsche *et al.* 2023). Interestingly, in a recent pediatric survey, more than 90% of cases of blastomycosis in children were caused by *B. gilchristii* compared with 56% of cases in adults (Frost *et al.* 2017).

Undoubtedly, genomic differences underpin the genetic segregation, reproductive isolation and different clinical manifestations of *B. dermatitidis* and *B. gilchristii*. Nonsynonymous substitutions, duplications, or deletions are examples of alterations to functional genetic components, such as protein coding genes or regulatory regions, which could impact fungal physiology. However, a large portion of eukaryotic genomes contains repetitive DNA with no known functional role in the development or physiology of the organism (Palazzo and Gregory 2014). This includes short tandem repeats and transposable elements (TE) such as retrotransposons and DNA transposons. TEs are found in almost all eukaryotic genomes but the relative TE content varies widely from <1% to >80% with large disparities in genomic TE content evident across all levels of taxonomic hierarchy of plants, animals, fungi, and protists (Arkhipova 2018). The evolutionary histories of most organisms likely involved bursts of transposition activity followed by phases of inactivity (Oggenfuss *et al.* 2021). TEs are less constrained by selective pressures (Palazzo and Gregory 2014; Arkhipova 2018) and have been shown to contribute substantially to the genome differences between fungal species (Grandaubert *et al.* 2014; Mat Razali *et al.* 2019; Lorrain *et al.* 2021) and even lineages of the same species (Faino *et al.* 2016; Oggenfuss *et al.* 2021).

Contrary to its ascription as “junk DNA”, transposable elements in particular can produce subtle alterations with profound evolutionary consequences (Mat Razali *et al.* 2019). TE invasions promote genome size expansion and function as agents of insertional mutagenesis. Counterbalancing the impact of TE invasion, homologous and illegitimate recombination mediate genome contraction in order to constrain genome size (Macas *et al.* 2015; Faino *et al.* 2016). Insertions in coding regions may alter protein structure and function while targeting regulatory regions can alter gene expression by exerting epigenetic control (Bourgeois and Boissinot 2019; Mat Razali *et al.* 2019). Although TE insertions generate genetic variability, the substrate for evolution, most insertions are selectively neutral or even slightly deleterious (Arkhipova 2018). Nevertheless, variation in the TE genomic landscape is purported to play a role in adaptive evolution, influencing host range, pathogenicity, and other aspects of fungal lifestyle (Mat Razali *et al.* 2019). For example in *Fusarium oxysporum*, TEs are present in the promoter regions of several genes located on the pathogenicity chromosome that are expressed during plant infection (Schmidt *et al.* 2013). The genomes of plant pathogens *Magnaporthe oryzae* and *Verticillium dahliae* contain lineage-specific regions abundant in both TEs and secreted proteins and genes that mediate virulence, host recognition and evasion of host defenses (Huang *et al.* 2014; Faino *et al.* 2016; Mat Razali *et al.* 2019). TE activity is postulated to drive accelerated evolution in these genome compartments which causes lineage-specific changes in virulence effectors such as the multiple independent lineage-specific losses of the host recognition determinant *AveI* in *V. dahliae* (Faino *et al.* 2016) and the presence/absence polymorphism of 6 avirulence *Avr*-genes necessary for rice plant host immune response across various strains of *M. oryzae* (Huang *et al.* 2014). TEs also shape genome structure by facilitating chromosomal rearrangements and acting as substrates for ectopic recombination (Bourgeois and Boissinot 2019; Mat Razali *et al.* 2019). TEs drive speciation by promoting reproductive isolation through genome reorganization causing genomic incompatibilities that can lead to pre- or post-zygotic barriers and hybrid inviability (Serrato-Capuchina and Matute 2018).

Despite the severity of blastomycosis and the importance of *B. dermatitidis* and *B. gilchristii* as fungal pathogens, only 4 *B. dermatitidis* and 1 *B. gilchristii* genome assemblies are publically available (Muñoz *et al.* 2015). Initial whole genome analysis and characterization of *B. dermatitidis* and *B. gilchristii* described highly expanded genomes with large isochore regions of low GC content. These regions had much lower gene density but were enriched in Gypsy long terminal repeat retrotransposons (LTR-RTs). Gypsy LTR-RTs were expanded in *B. gilchristii* strain SLH14081 compared to *B. dermatitidis* strain ER-3, contributing to an 8.8 Mb genome assembly size difference (Muñoz *et al.* 2015). In this study, we have provided additional phylogenomic analysis of *B. dermatitidis* and *B. gilchristii* and expanded the genome characterization with 2 additional de novo assembled whole genome sequences of each species. In addition to confirming that the *B. gilchristii* genome is on average 8.6 Mb larger than that of *B. dermatitidis*, we compared average nucleotide identity (ANI), proteome functional classifications, and differing LTR-RT landscapes, providing insight into the role of repetitive elements in the evolution of these cryptic species.

## Materials and methods

### Isolates

Forty isolates were randomly selected from among those cultured from Ontario patient specimens received by Public Health Ontario's Laboratory between February 2016 and July 2022. Using multilocus sequence analysis (Brown *et al.* 2013), 22 isolates were identified as *B. gilchristii* and 18 as *B. dermatitidis* (Supplementary Table 1).

### Whole genome sequencing and phylogenomic analysis

Illumina short-read whole genome sequencing was performed on 20 *B. gilchristii* isolates and 16 *B. dermatitidis* isolates (Supplementary Table 1). Briefly, DNA was extracted using the DNeasy PowerSoil Pro kit (Qiagen, Germantown, MD, USA). Libraries were prepared using the Illlumina DNA prep kit (Illumina, San Diego, CA, USA) and sequenced on a NextSeq 550 (Illumina) at 2 × 150 bp read length. Fastq files are available in the NCBI Sequence Read Archive (SRA) under Bioproject accession number PRJNA890593 (Supplementary Table 1).

Two isolates of each species were selected for whole genome sequencing using linked reads. Isolates 22,281 (*B. dermatitidis*) and 23,019 (*B. gilchristii*) were derived from respiratory specimens, bronchoalveolar lavage and sputum, respectively; while 23,166 (*B. dermatitidis*) and 22,264 (*B. gilchristii*) were cultured from tissue. For these isolates, DNA was extracted from fungal material cultured in SAB broth for 5 days at 28°C and 200 rpm. After filtering from broth using vacuum filtration and a sterile filter unit, fungal material was crushed in liquid nitrogen and processed using the MagAttract HMW DNA kit (Qiagen). Next generation sequencing was performed at Canada's Genomic Enterprise (CGEn), The Centre for Applied Genomics (Toronto, ON, Canada) using Chromium Genome library preparation v2 kit (10X Genomics, Pleasanton, CA, USA). Libraries were sequenced on a HiSeq 2500 (Illumina) at 2 × 150 bp read length following the manufacturer's instruction (Supplementary Table 1). For downstream analyses of de novo assembled genomes, fastq files were assembled using supernova software v 2.1.1 (Weisenfeld *et al.* 2018). Since haploid genomes were sequenced, pseudohap assemblies were used for subsequent analyses requiring de novo assembled genomes. Files are available under NCBI BioProject PRJNA890593 (Supplementary Table 1).

### Species tree

For 3 *B. dermatitidis*, 3 *B. gilchristii*, 7 *B. emzantsi*, 1 *Blastomyces parvus*, 14 *B. percursus*, and 1 *Blastomyces silverae* for which genome assemblies were available (Supplementary Table 1), genomes were masked using RepeatMasker v4.1.1 (Smit *et al.* 2013–2015) with repeat libraries generated using RepeatModeler v2.0. (Flynn *et al.* 2020). Proteins were predicted using funannotate (https://github.com/nextgenusfs/funannotate) with BUSCOs from *Histoplasma capsulatum* and protein evidence from UniProt (The UniProt Consortium 2023). A species tree was generated using OrthoFinder (Emms and Kelly 2019) using proteins predicted by funannnotate. Briefly, the consensus species tree was inferred using STAG (Emms and Kelly 2018) from gene trees generated for orthogroups where all individuals were present. The species tree was rooted using STRIDE (Emms and Kelly 2017).

### Whole genome single-nucleotide variant phylogenomic analysis

Whole genome single-nucleotide variant (SNV) phylogenomic analysis was conducted using the MycoSNP pipeline (Bagal, Phan, *et al.* 2022) with reference genome *B. gilchristii* SLH14081 (GCF_000003855.2) using sequence data generated above and an additional 22 *B. dermatitidis* and 5 *B. gilchristii* raw fastq files downloaded from the NCBI SRA (Supplementary Table 1). Prior to MycoSNP analysis, the raw fastq files from the 4 genomes sequenced with the Chromium Genome library preparation (22,264, 22,281, 23,019, 23,166) were processed with the longranger basic pipeline (10X Genomics) for barcode handling and removal. Maximum-likelihood (ML) phylogenetic analysis of concatenated whole genome SNVs generated by MycoSNP was conducted in IQ-TREE 2 with ascertainment bias correction, ModelFinder plus, and 1,000 ultrafast bootstrap approximations (Nguyen *et al.* 2015; Kalyaanamoorthy *et al.* 2017; Hoang *et al.* 2018; Minh *et al.* 2020). The tree was depicted in R v4.2.1 (R Core Team (2022)) using ggtree (Yu 2020).

### Comparison of *B. dermatitidis* and *B. gilchristii*

For an in-depth comparison of *B. dermatitidis* and *B. gilchristii* genomes, we utilized the 4 de novo assembled genomes generated by the Chromium linked-read technology and *B. gilchristii* SLH14081 and *B. dermatitidis* ER-3 (Supplementary Table 1), which represent the most complete genome assemblies available for each of these species.

#### ANI comparisons and protein functional annotation

To assess inter- and intraspecific ANI of *B. dermatitidis* and *B. gilchristii*, pairwise comparisons of these genomes was performed using OrthoANI (Lee *et al.* 2016). Protein predictions were made using funannotate as described above with additional expressed sequence tag (EST) evidence generated from RNA reads from *B. dermatitidis* ATCC 26199 (PRJNA185598) assembled using Trinity (Grabherr *et al.* 2011). Funannotate functional annotation incorporated protein functional predictions from InterProScan v5.59-91.0 (Jones *et al.* 2014; Blum *et al.* 2021) and Phobius (Käll *et al.* 2007). Comparative analysis was performed using the funannotate compare function with Chi-square tests and Benjamini–Yekutieli correction for multiple comparisons (Benjamini and Yekutieli 2001) used to assess differences in functional protein counts between *B. dermatitidis* and *B. gilchristii* isolates.

### Repetitive sequence analysis

#### LTR-RT library construction

Using genomes ER-3 and SLH14081, candidate LTR-RT were identified using LTR_Finder (Xu and Wang 2007) and LTRharvest (Ellinghaus *et al.* 2008) using default parameters. Using these candidates, LTR_retriever (Ou and Jiang 2018) was used to generate a set of 232 full length, intact LTR-RTs in ER-3 and 50 in SLH14081. TEsorter (Zhang *et al.* 2022) employing the GyDB (Llorens *et al.* 2011) was used to detect functional domains and to classify each of the full length, intact LTR-RTs as Gypsy, Copia, or unknown. The 2 sets of LTR-RTs were combined to create a redundant *Blastomyces* LTR-RT library. Using CD-HIT (Huang *et al.* 2010), the redundant *Blastomyces* LTR-RT library was clustered according to the 80–80–80 rule (80 bp long, 80% identity, 80% minimal alignment coverage for the longer sequence) (Wicker *et al.* 2007). A nonredundant *Blastomyces* LTR-RT library was constructed from representative sequences of each cluster.

#### Repetitive sequence annotation

RepeatMasker was run on all isolates using the nonredundant *Blastomyces* LTR-RT library with parameters -no_is -norna -nolow -div 40 -cutoff 225. Then, non-LTR-RT repetitive sequences or each genome were identified using RepeatModeler on the *Blastomyces* LTR-RT masked genomes from the previous step. The nonredundant *Blastomyces* LTR-RT library was combined with each isolate-specific RepeatModeler library for a final round of RepeatMasker to include low diversity and simple repeats as well transposable elements (TEs) including LTR-RTs. The statistical significance of comparisons genomic size data and element count data were evaluated by 2-tailed *t*-test and Chi-square goodness-of-fit test, respectively, with Benjamini–Yekutieli correction for multiple comparisons (Benjamini and Yekutieli 2001).

#### Annotation of solo-LTRs and complete, truncated, and nested LTR-RTs

We used the software program REANNOTATE (Pereira 2008) to detect solo-LTRs and complete (interrupted or not interrupted), truncated and nested LTR-RTs from all study isolates using the RepeatMasker output from a redundant *Blastomyces* LTR-RT library scan with parameters -no_is -norna -nolow -div 40 -cutoff 225 and a “fuzzy” file linking equivalent LTR-RT elements based on identical clustering of INT and LTR elements by CD-HIT.

#### Repetitive sequence divergence and dating

The output (.align files) from the final round of RepeatMasker was further parsed using scripts from https://github.com/4ureliek/Parsing-RepeatMasker-Outputs (Kapusta and Suh 2017) to examine the divergence of repetitive sequence elements compared to the family consensus sequence and employing a fungal substitution rate of 1.05 × 10^−9^ nucleotides/site/year (Castanera *et al.* 2016) to estimate element age.

The insertion ages of intact LTR-RTs of ER-3 and SLH14081 were calculated in LTR_retriever by estimating the divergence time of the 3′ and 5′ LTR regions using the Jukes-Cantor model for noncoding sequences (Jukes and Cantor 1969; Ou and Jiang 2018) and applying the aforementioned fungal substitution rate.

#### Phylogenetic analysis of reverse transcriptase (RT) domains

Genomic TE sequences were extracted from all 6 study isolates using the output from the final round of RepeatMasker and the out2seqs scripts from the TEsorter package (Zhang *et al.* 2022). Six-frame translations of the DNA TE sequences were conducted using transeq from EMBOSS (Rice *et al.* 2000) using the –clean parameter to specify “X” for stop codons. TEsorter and the GyDB were used to classify the translated TE sequences using parameters -st prot -db gydb -p 20 -cov 50 -eval 1e-5 and extract the RT domains. Sequences of Copia or select Gypsy families from each of the 6 study isolates were combined then analyzed by phylogenetic comparison employing MAFFT for alignment (Katoh and Standley 2013) and IQ-TREE 2 for ML phylogenetic analysis employing ModelFinder plus and 1,000 ultrafast bootstrap approximations (Nguyen *et al.* 2015; Kalyaanamoorthy *et al.* 2017; Hoang *et al.* 2018; Minh *et al.* 2020). Trees were depicted in R v4.2.1 (R Core Team (2022)) using ggtree (Yu 2020).

#### Syntenic comparison of the *MAT* locus

The *MAT* locus of SLH14081 is located on supercontig NW_003101669.1:991747-1048538 bounded by the AP endonuclease 2 (*AP2*) and SlaB genes. For each isolate, *MAT* locus region was subset from the assembly contigs based on blast searches against the SLH14081 *MAT* locus and single-copy genes *APN2* and *SlaB*. Syntenic comparison of the *MAT* locus was visualized using the Artemis comparison tool (Carver *et al.* 2005) using comparison files generated from pairwise blast searches. Repetitive element annotation was added from the final round of RepeatMasker using the nonredundant *Blastomyces* LTR-RT library combined with each isolate-specific RepeatModeler library.

## Results

### Species tree and whole genome SNV phylogenomic analysis demonstrate that *B. dermatitidis* and *B. gilchristii* are genetically distinct

**Fig. 1.**
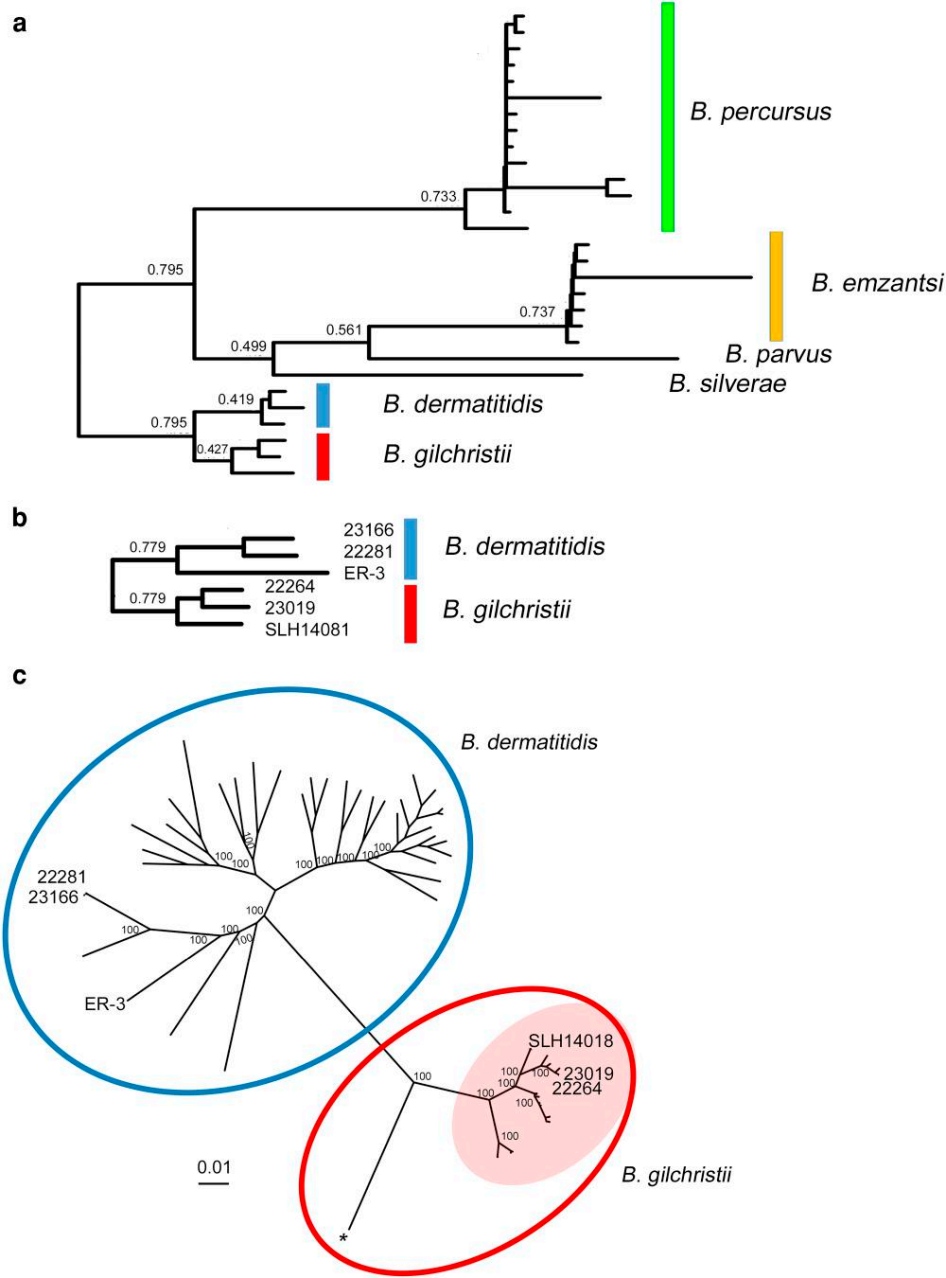
a) Species tree of the genus *Blastomyces* including *B. dermatitidis* (3), *B. gilchristii* (3), *B. emzantsi* (7), *B. parvus* (1), *B. percursus* (14), and *B. silverae* (1). The species tree was generated by OrthoFinder based on 5,238 orthogroups for which all individuals were present. Branch support values for each bipartition indicate the proportion of individual orthogroup species trees that contained that bipartition. b) OrthoFinder species tree generated for *B. dermatitidis* and *B. gilchristii* based on 6,962 orthogroups. c) Phylogenomic analysis of 268,539 whole genome SNVs from 40 *B. dermatitidis* and 28 *B. gilchristii* isolates by ML using the transversion model with ascertainment bias correction. A single *B. gilchristii* isolate from Quebec (*) is genetically divergent from other *B. gilchristii* isolates from Ontario, Minnesota and Quebec (opaque circle). The tree is drawn to scale with branch lengths measured in the number of substitutions per site. The percentage of trees of 1,000 ultrafast bootstrap approximations in which the associated taxa clustered together is shown next to the nodes.

A species tree based on orthogroups constructed by OrthoFinder using protein sequences predicted from genome assemblies of 3 *B. dermatitidis*, 3 *B. gilchristii*, 7 *B. emzantsi*, 1 *B. parvus*, 14 *B. percursus*, and 1 *B. silverae* clearly delineated these 6 *Blastomyces* spp. (Fig. 1a). Based on 5,238 orthogroups, cryptic species *B. gilchristii* and *B. dermatitidis* were more closely related with lower branch support values (0.427, 0.419, respectively) compared to the other species *B. percursus* (0.733) and *B. emzantsi* (0.737). However, species tree branch support values for *B. dermatitidis* and *B. gilchristii* were higher (0.779) when analyzed independent of the other *Blastomyces* spp. using an expanded set of 6,962 orthogroups common to *B. dermatitidis* and *B. gilchristii* (Fig. 1b).

Whole genome SNV phylogenomic analysis encompassing 268,539 sites separated *B. dermatitidis* (*n* = 40) and *B. gilchristii* (*n* = 28) into 2 phylogenetically distinct groups (Fig. 1c). The average interspecies genetic distance was 1.185 ± 0.072 which was greater than the average intraspecies genetic distance for either *B. dermatitidis* (0.304 ± 0.160) or *B. gilchristii* (0.103 ± 0.122). Genetic diversity among *B. gilchristii* isolates was lower than among *B. dermatitidis* isolates. Of note, a single isolate from Quebec was genetically divergent from other isolates from Ontario, Minnesota, and Quebec (Fig. 1c).

### *B. dermatitidis* and *B. gilchristii* genomes were different sizes with reduced ANI but similar gene content

To further characterize the genetic differences between *B. dermatitidis* and *B. gilchristii*, we utilized 4 genomes de novo assembled from linked-read sequences representing 2 clinical isolates of *B. dermatitidis* (22,281 and 23,166) and 2 clinical isolates of *B. gilchristii* (22,264 and 23,019) isolates together with 2 well-characterized reference strains *B. dermatitidis* ER-3 and *B. gilchristii* SLH14081 (Muñoz *et al.* 2015). Together, these genomes confirmed the previously documented 8.6 Mb average genome size difference (Muñoz *et al.* 2015) between *B. gilchristii* (73.3–75.4 Mb) and *B. dermatitidis* (64.9–66.6 Mb) (Table 1). The percentage of Onygenales universal single copy orthologs (BUSCOs) detected in the genome assemblies was high (97.3–98.5%) (Table 1). The GC content of the genomes ranged from 35.8–37.3% (Table 1), however, the GC frequency distribution of the short-read sequences exhibited a bimodal distribution as previously described (Supplementary Fig. 1) (Muñoz *et al.* 2015). Furthermore, the ANI between species was 96.6%, while the intraspecies ANI ranged from 97.1–99.5% for *B. dermatitidis* and 98.7–99.4% for *B. gilchristii* (Supplementary Table 2). *B. gilchristii* genomes had more funannotate-predicted genes (8,467 ± 170) than *B. dermatitidis* genomes (8,218 ± 178), although this difference was not significant (*P* = 0.155). Despite these differences, comparison of counts of InterProScan classifications revealed no significant differences between the species (Supplementary Table 3).

**Table 1.** Genome characteristics.

Isolate	Total assembly length	No. scaffolds	Scaffold N_50_	% BUSCOs in genome	GC content (%)	Genes
*B. dermatitidis*						
ER3	66.61 Mb	25	5.55 Mb	98.4%	37.1%	8,175
22,281	64.96 Mb	2,550	0.080 Mb	98.3%	37.2%	8,066
23,166	64.88 Mb	2,739	0.071 Mb	98.3%	37.3%	8,414
*B. gilchristii*						
SLH14081	75.40 Mb	100	2.44 Mb	98.5%	35.8%	8,329
22,264	73.53 Mb	2,981	0.063 Mb	98.1%	35.8%	8,416
23,019	73.25 Mb	3,475	0.048 Mb	97.3%	35.8%	8,657

### Gypsy LTR-RTs distinguish *B. dermatitidis* and *B. gilchristii* genomes

**Fig. 2.**
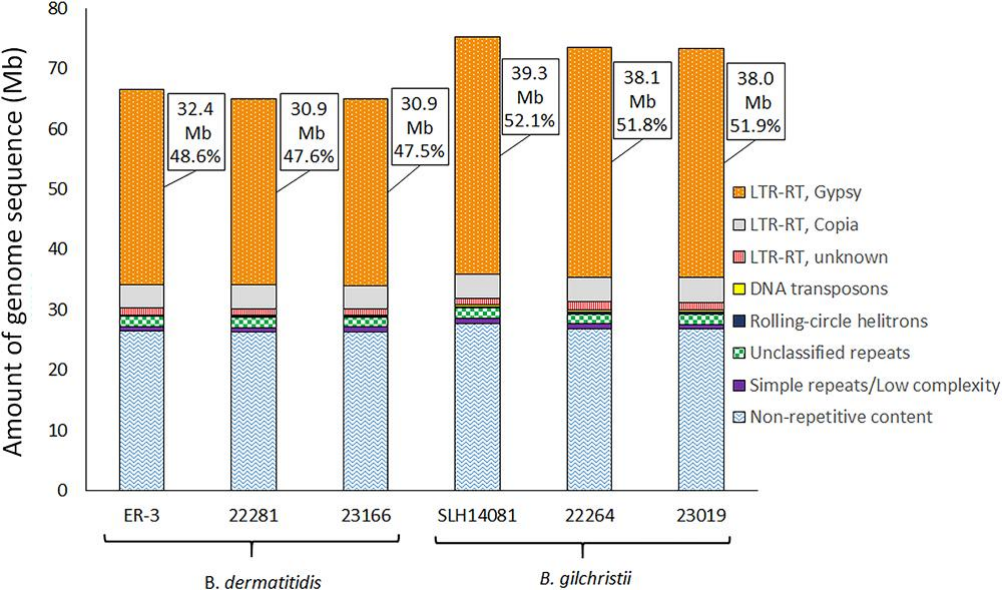
Amount of genome sequence contribution for classes of repetitive elements and non-repetitive content of *B. dermatitidis* and *B. gilchristii* genomes. The quantity (Mb) and percentage of each genome comprised of Gypsy LTR-RTs is indicated.

A large proportion of the *Blastomyces* genomes was composed of repetitive elements, ranging from 59.4–60.3% (38.6–40.2Mb) of the *B. dermatitidis* genomes and 63.0–63.6% (46.6–47.5Mb) of the *B. gilchristii* genomes. The vast majority of the repetitive content was attributed to LTR-RTs, with less than 5% of the genomes ascribed to other transposable elements and simple repeats. Gypsy LTR-RTs were the predominant LTR-RT superfamily, comprising 47.5–52.1% of the genomes (Table 2; Fig. 2). Furthermore, the statistically significant 8.6 Mb average size difference between *B. dermatitidis* and *B. gilchristii* genomes (*P* = 0.0092) was attributed largely to LTR-RTs. LTR-RTs and specifically Gypsy LTR-RTs showed a corresponding 7.5 Mb and 7.1 Mb statistically significant size difference, respectively, between the species genomes (*P* = 0.0043; 0.0044) (Table 2).

**Table 2.** Characterization of repetitive elements in *Blastomyces* genomes including sequence contributions of repetitive elements and TE superfamilies and number of LTR-RT elements characterized as complete, nested, truncated or solo-LTRs.

	*B. dermatitidis*				*B. gilchristii*				
	ER-3	22,281	23,166	Avg. Bd	SLH14081	22,264	23,019	Avg. Bg	*P*_adj_
Genome size	66.61	64.96	64.88	65.48	75.40	73.53	73.25	74.06	**0**.**0092**
Total repeat content	40.19	38.69	38.59	39.16	47.70	46.67	46.49	46.95	**0**.**0110**
	(60.3%)	(59.6%)	(59.5%)	(59.8%)	(63.3%)	(63.5%)	(63.5%)	(63.4%)	
LTR-RT	37.51	35.88	35.83	36.41	44.65	43.59	43.42	43.89	**0**.**0043**
	(56.3%)	(55.2%)	(55.2%)	(55.6%)	(59.2%)	(59.3%)	(59.3%)	(59.3%)	
Gypsy	32.40	30.92	30.85	31.39	39.26	38.11	38.04	38.47	**0**.**0044**
	(48.6%)	(47.6%)	(47.5%)	(47.9%)	(52.1%)	(51.8%)	(51.9%)	(51.9%)	
Copia	3.98	3.85	3.84	3.89	4.05	4.16	4.09	4.10	0.0981
	(6.0%)	(5.9%)	(5.9%)	(5.9%)	(5.4%)	(5.7%)	(5.6%)	(5.5%)	
DNA transposon	0.20	0.23	0.22	0.22	0.35	0.35	0.35	0.35	**0**.**0221**
	(0.3%)	(0.4%)	(0.3%)	(0.3%)	(0.5%)	(0.5%)	(0.5%)	(0.5%)	
RC-helitron	0.05	0.06	0.14	0.08	0.10	0.29	0.29	0.23	0.5028
	(0.1%)	(0.1%)	(0.2%)	(0.1%)	(0.1%)	(0.4%)	(0.4%)	(0.3%)	
Unclassified	1.67	1.75	1.61	1.68	1.84	1.65	1.70	1.73	1
	(2.5%)	(2.7%)	(2.5%)	(2.6%)	(2.4%)	(2.2%)	(2.3%)	(2.3%)	
Simple/low complex	0.75	0.77	0.78	0.77	0.76	0.77	0.77	0.77	1
	(1.1%)	(1.2%)	(1.2%)	(1.2%)	(1.0%)	(1.1%)	(1.1%)	(1.0%)	
No. LTR-RT elements	22,075	25,715	25,863	24,551	26,608	30,427	30,573	29,203	**<0**.**0001**
No. complete LTRs*^a^*	540	329	332	400	476	409	392	426	0.2896
No. nested LTR-RTs	13,863	11,253	11,164	12,093	17,087	14,114	13,562	14,921	**<0**.**0001**
No. truncated LTR-RTs	20,001	23,648	23,810	22,486	24,165	27,759	27,899	26,608	**<0**.**0001**
No. solo-LTRs	2,585	3,092	3,100	2,926	3,329	3,659	3,704	3,564	**<0**.**0001**

*P*_adj_ values of statistically significant comparisons between *B. dermatitidis* and *B. gilchristii* are in bold. All sizes are in megabases (Mb).

^*a*^Includes interrupted and non-interrupted LTR-INT-LTR combinations.

**Fig. 3.**
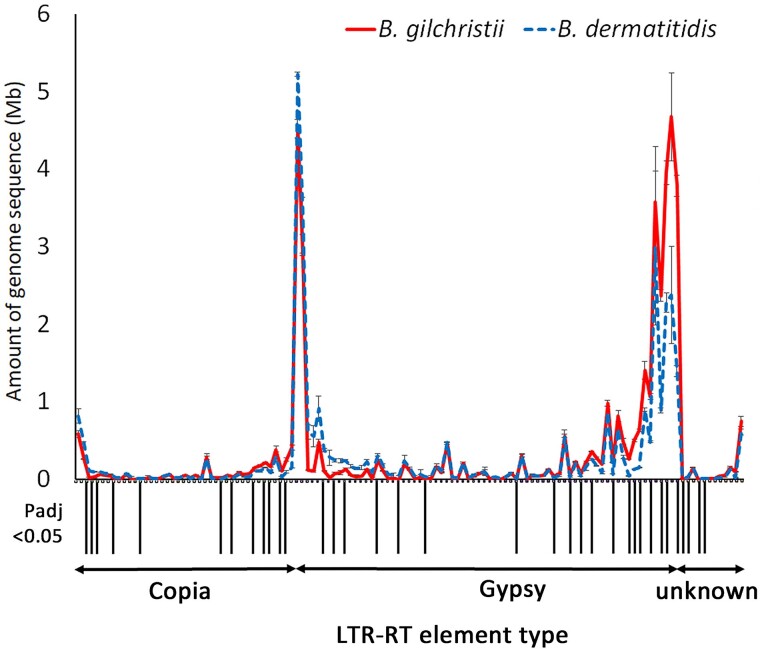
Average genome sequence contribution of 125 LTR-RT (72 Gypsy, 41 Copia, 12 unknown) elements to *B. dermatitidis* and *B. gilchristii* genomes. Analysis included elements with an average of at least a 10,000 bp genome contribution in at least 1 species. Elements with a statistically significant difference in the amount of genome sequence contribution between the 2 species are designated with black bars along the *x*-axis.

To facilitate LTR-RT annotation and comparison, we constructed a *Blastomyces* nonredundant LTR-RT library based on 232 intact LTR-RTs from *B. dermatitidis* ER-3 and 50 intact LTR-RTs of *B. gilchristii* SLH14081 which was used for identification of LTR-RT elements in each for the 6 genomes. Examination of LTR-RT elements comprising on average >10,000 bp in the genome showed that *B. dermatitidis* and *B. gilchristii* possessed the same LTR-RT elements but the quantities of several elements differed between the species. Of the 125 elements examined, statistically significant differences were observed among 19 Gypsy, 12 Copia, and 4 unknown LTR-RT elements. Sixteen elements were on average statistically more abundant in *B. dermatitidis* contributing an average of 0.97 million more bases to the *B. dermatitidis* genomes compare to the *B. gilchristii* genomes (Fig. 3). Conversely, 19 elements were statistically more abundant in *B. gilchristii* adding on average 7.97 million more bases to the *B. gilchristii* genomes compared to the *B. dermatitidis* genomes (Fig. 3). Of note, the greatest size differential was observed with elements Bder197_INT, Bgil46_INT, and Bder129_INT, which exhibited on average 1.48, 1.68, and 2.39 million more base pairs, respectively, in the *B. gilchristii* genomes compared to the *B. dermatitidis* genomes.

LTR-RTs were additionally characterized by estimating the number of complete (interrupted and non-interrupted), nested, and truncated LTR-RTs and solo-LTRs in each of the *Blastomyces* genomes. This approach involved re-annotating the LTR-RTs in the genomes using the redundant *Blastomyces* LTR-RT library, which contains information to associate the LTR and internal components of the LTR-RTs, and a linker file to identify equivalent LTR-RT elements based on clustering of INT and LTR elements by CD-HIT. Between 22,075 and 30,573 LTR-RT elements were annotated per genome (Table 2). Few elements comprised complete (interrupted and non-interrupted) LTR-RTs. Instead, an overwhelming number of LTR-RT elements were present as nested and/or truncated. In addition, the number of solo-LTRs per genome ranged from 2,585–3,704. Statistically significantly more nested and truncated LTR-RTs and solo-LTRs were detected in the *B. gilchristii* genome compared to *B. dermatitidis* (Table 2).

### *Blastomyces* TEs, including LTR-RTs, are highly divergent

**Fig. 4.**
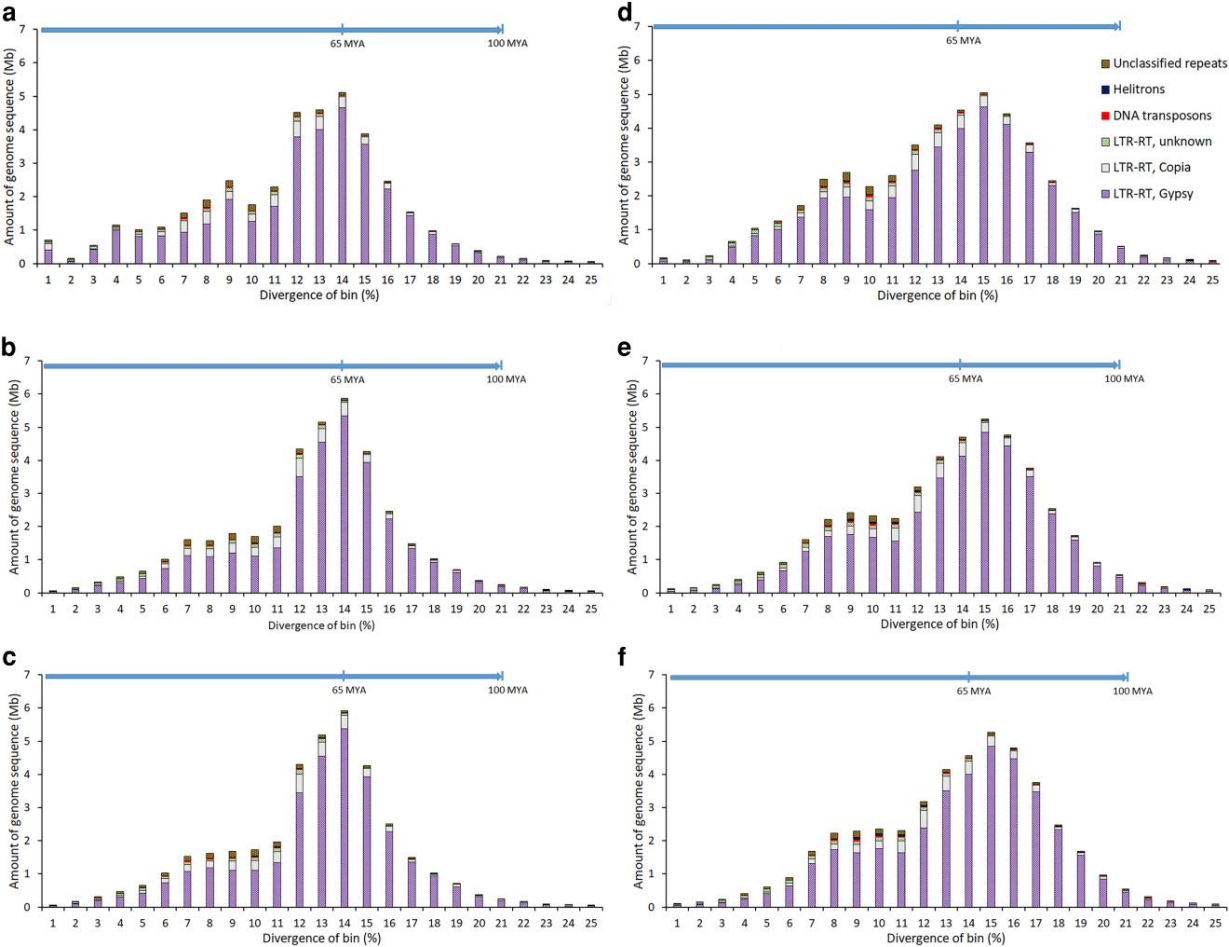
Divergence landscape of TEs of *B. dermatitidis* ER-3 a) 22,281, b) and 23,166 c) and *B. gilchristii* SLH14081, d) 22,264, e), and 23,019 f). Stacked histograms express the amount of genome sequence categorized into bins of percent divergence of TE family members compared to their respectively library consensus sequence. Element dating (million years ago, MYA) is calculated using a fungal substitution rate of 1.05 × 10^−9^ nucleotides/site/year (Castanera *et al.* 2016).

Divergence of TEs was tabulated by comparing TE family members to their respective consensus sequences. The TE landscape graphs presented as stacked histograms of percent divergence suggested that *Blastomyces* TEs, including LTR-RTs, were highly diverged. Across all genomes, more than 50% of TE content was highly divergent (≥12%), while less than 10% exhibited a high degree of similarity (≤5% divergence) (Fig. 4). Applying a fungal substitution rate of 1.05 × 10^−9^ nucleotides/site/year (Castanera *et al.* 2016) suggested that the vast majority of *Blastomyces* TEs were ancient (Fig. 4) and predate the separation of *B. dermatitidis* and *B. gilchristii* estimated at 1.9 million years ago (MYA) (95% HPD 0.5–4.4 MYA) (McTaggart *et al.* 2016). The peaks of the histograms of the TE divergence landscapes of the *B. dermatitidis* (Fig. 4, a–c) isolates were shifted left compared to those of *B. gilchristii* (Fig. 4, d–f), suggesting that the *B. gilchristii* TEs were generally more divergent compared to those of *B. dermatitidis*.

**Fig. 5.**
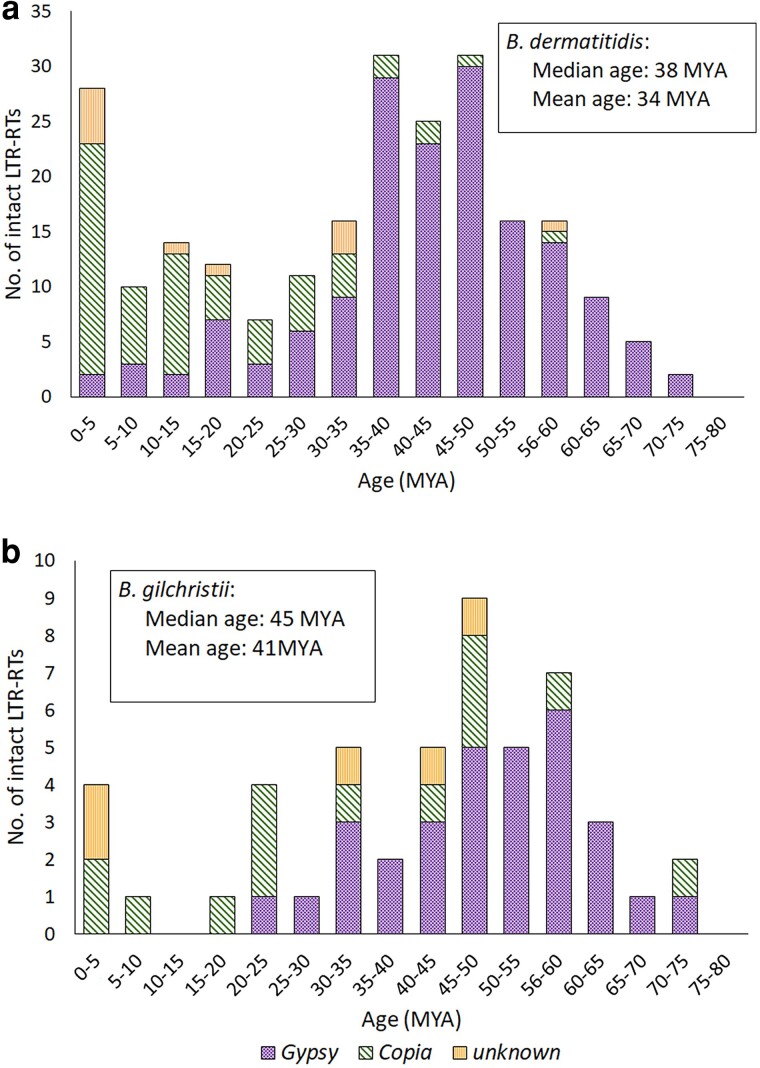
Estimated insertion age (million years ago, MYA) of a) 232 intact LTR-RTs of *B. dermatitidis* ER-3 and b) 50 intact LTR-RTs of *B. gilchristii* SLH14081. Insertion age is calculated based on the sequence divergence between 5′ and 3′ LTR regions of the elements.

We also estimated the insertion ages of intact LTR-RTs in the 2 most contiguous genomes, *B. dermatitidis* ER-3 and *B. gilchristii* SLH14081. Since the 3′ and 5′ LTR regions of an LTR-RT are identical at the time of insertion, the age of an element can be estimated based on the divergence of these sequences (Ou and Jiang 2018; Rao *et al.* 2018). Of note, 232 intact LTR-RTs were identified in *B. dermatitidis* ER-3 while only 50 were detected in the *B. gilchristii* SLH14081 genome. The insertion ages of the intact LTR-RTs of *B. gilchristii* were on average older (median: 45 MYA; mean: 41 MYA) than that of *B. dermatitidis* intact LTR-RTs (median: 38 MYA, mean: 34 MYA) (Fig. 5). Furthermore, there were 28 intact LTR-RTs (2 Gypsy, 21 Copia, 5 unknown) with an insertion age < 5 MYA in the *B. dermatitidis* ER-3 genome but only 4 (2 Copia, 2 unknown) in *B. gilchristii* SLH14081 (Fig. 5). Based on TEsorter analysis, none of the intact LTR-RTs in either species contained all of the functional domains required for transposition.

### Phylogenetic analysis of reverse transcriptase domains of *B. dermatitidis* and *B. gilchristii* LTR-RT families cluster together

**Fig. 6.**
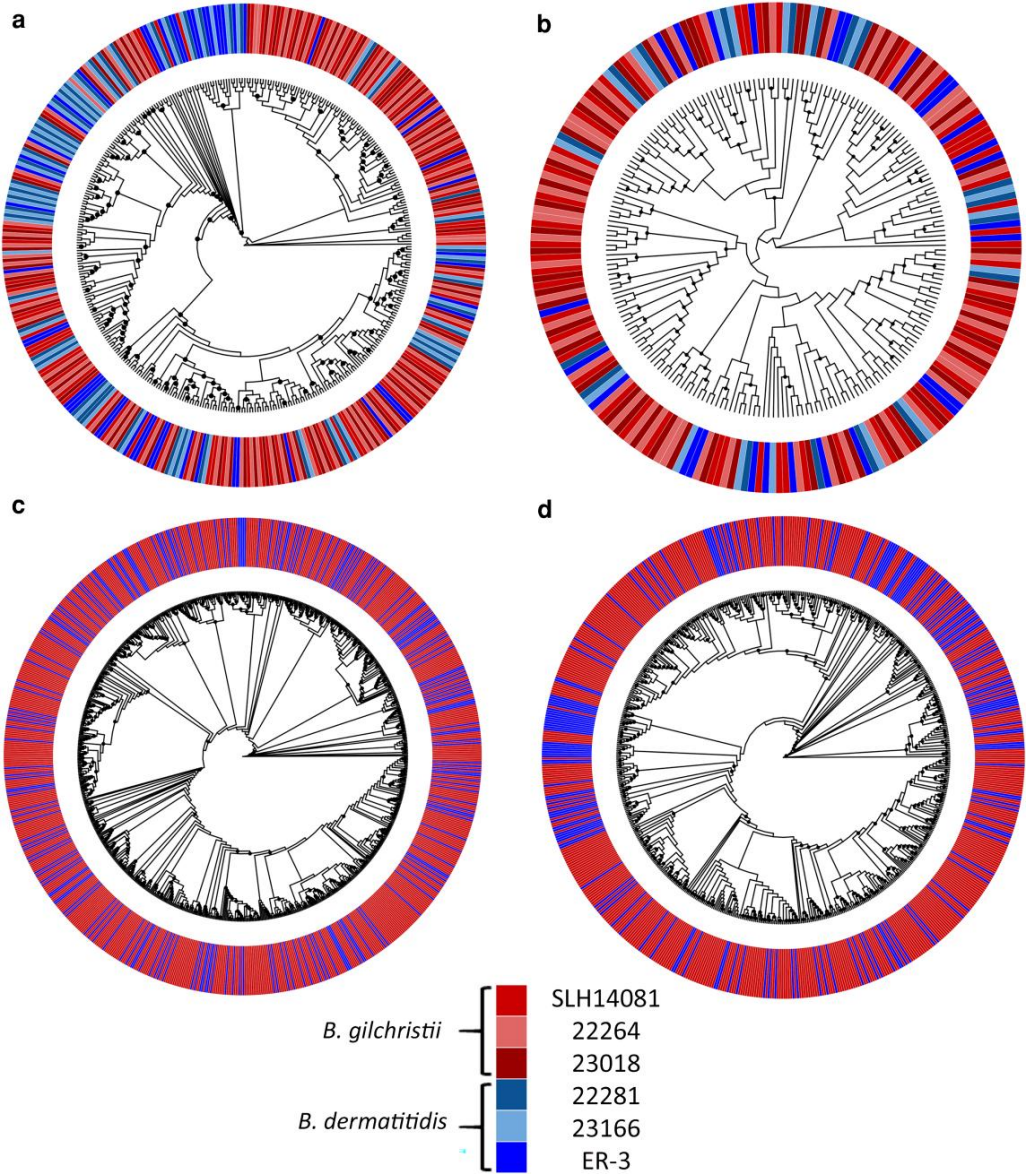
Cladograms showing ML phylogenetic relationships inferred from alignments of reverse transcriptase domains of Copia LTR-RT elements a) and 3 families of Gypsy LTR-RT elements Bgil46_INT b), Bder129_INT, c) and Bder197_INT d). Analyses of Copia and Gypsy Bgil46_INT elements employed reverse transcriptase domains from all 6 isolates (a and b). For simplicity, analyses of Gypsy Bder129_INT and Bder197_INT utilized reverse transcriptase domains from SLH14081 and ER-3 only (c and d).

Phylogenetic analysis was used to analyze the evolutionary relationships between reverse transcriptase domains of the LTR-RTs. Due to the sheer number of LTR-RT elements, phylogenetic analysis was only feasible on select subsets of elements. Copia LTR-RT reverse transcriptase domains from all isolates were selected for analysis (Fig. 6a) because there were a relatively large number of young (<5 MYA), intact Copia LTR-RT elements present in ER-3 and SLH14081. As well, we analyzed reverse transcriptase domains from 3 sets of Gypsy elements [Bgil46_INT (Fig. 6b), Bder129_INT (Fig. 6c), Bder197_INT (Fig. 6d)] previously shown to be present in statistically significantly greater quantities (Mbp) in the *B. gilchristii* genomes compared to the *B. dermatitidis* genomes. For all sets of reverse transcriptase domains examined, the phylogenies did not delineate according to species. Although some small species-exclusive clades were observed, most clades contained sequences from both *B. dermatitidis* and *B. gilchristii* (Fig. 6).

### Presence of TEs around the mating-type locus is variable

*Blastomyces* spp. are heterothallic with the mating type determined by the presence of either the alpha-box (*MAT1-1*) or HMG domains (*MAT1-2*) at the *MAT* locus, located between the *APN2*, cytochrome c oxidase subunit 6a *(COX13)* and *SlaB* genes (Li *et al.* 2013). Syntenic analysis of the *MAT* locus revealed a size variation at the *MAT* locus due to the presence of transposable element sequence content as previously documented (Li *et al.* 2013) with *B. gilchristii* isolates possessing a much larger *MAT* locus due to the presence of Gypsy LTR-RT sequence content compared to the *B. dermatitidis* isolates (Supplementary Fig. 2).

## Discussion

In this study, we provide a genomic analysis and comparison of *B. dermatitidis* and *B. gilchristii*, the causative agents of the serious fungal disease blastomycosis, complementing the paucity of publically available genome assemblies and genomic studies available for these organisms (Muñoz *et al.* 2015; Carignan *et al.* 2021; Bagal, Ireland, *et al.* 2022). Both a species tree based on orthogroups and whole genome SNV phylogenomic analysis demonstrate the phylogenetic separation between *B. dermatitidis* and *B. gilchristii*. However, there were no significant differences in gene functional classifications, which is consistent with the identical morphologies of these cryptic species. The most substantial genomic different between the 2 species is the quantity of TE content, particularly LTR-RT content, yielding a *B. gilchristii* genome that is ∼8 Mb larger than the *B. dermatitidis* genome. We demonstrate that the LTR-RTs are relatively ancient and posit that the 2 species experienced different evolutionary trajectories of genome contraction which facilitated and/or maintained reproductive isolation.

Originally differentiated by MLST of 7 nuclear loci (Brown *et al.* 2013), we show a phylogenetic separation between *B. dermatitidis* and *B. gilchristii* using more robust genetic methods, namely a consensus *Blastomyces* species tree generated from phylogenetic analysis of individual orthogroups and phylogenomic analysis of whole genome SNV data from a dataset of 68 sequenced *B. dermatitidis* and *B. gilchristii* isolates. Average interspecies genetic distance based on the SNV data was greater than intraspecies genetic distances, demonstrating the genetic differentiation and divergence between species and supporting their status as separate, albeit cryptic species. Although genetic diversity among *B. gilchristii* isolates was lower than among *B. dermatitidis* isolates, a single isolate from Quebec suggests that the genetic diversity among *B. gilchristii* isolates may prove to be greater as more sequence data from isolates from geographically diverse location becomes available.

To facilitate more detailed genomic comparison and analysis, we sequenced and assembled genomes of 2 *B. dermatitidis* (22,281, 23,166) and 2 *B. gilchristii* (22,264, 23,019) isolates using Illumina short-read sequencing with the 10X Genomics’ Chromium technology, which uniquely barcodes fragments of the next generation sequencing libraries to aid in de novo assembly (Weisenfeld *et al.* 2018). Although the 10X Genomics’ Chromium libraries produced genomes that were more fragmented than the Sanger-sequenced genomes of ER-3 and SLH14081 (Muñoz *et al.* 2015), the genome sizes were similar and the representation of core eukaryotic genes was high, suggesting that the genomes are very nearly complete. Illumina linked reads have been used to generate high quality genome assemblies for other species (Ott *et al.* 2018; Ozerov *et al.* 2018; Wang *et al.* 2020). Although long-read sequencing technologies such as PacBio and Nanopore are an attractive alternative to Illumina linked-read assemblies, they have both a higher cost and error rate. In the future, a hybrid approach involving both short-read and long-read sequencedata would greatly assist the generation of more complete genome assemblies for *Blastomyces* spp.

The genomes of *B. dermatitidis* and *B. gilchristii* were distinctly different based on genome size and ANI. We confirmed a notable genome size difference between species, with *B. gilchristii* genomes (73.3–75.4 Mb) approximately 8 Mb larger than *B. dermatitidis* genomes (64.9–66.6 Mb). We also verified the bimodal GC frequency distribution of the short-read fragments of both species suggestive of large isochore-like genomic regions of high and low GC content (Muñoz *et al.* 2015). Likewise, intraspecies ANI were higher than interspecies ANI values. The interspecies ANI values (96.53–96.60%) were slightly higher than the recommended cutoff of 95–96% for species demarcation (Lee *et al.* 2016), which is consistent with their status as cryptic species. Diminished whole genome synteny between *B. dermatitidis* ER-3 and *B. gilchristii* SLH14081 was described by (Muñoz *et al.* 2015), largely confined to the GC-poor, repeat-rich regions of the genome.

Gene annotation and functional classification revealed that *B. dermatitidis* and *B. gilchristii* displayed no significant differences in gene functional quantification, which was expected given their similar morphologies, ecology, and clinical manifestations. Although not examined in this study, phenotypic variation between species can be caused by genetic alterations to regulatory regions that impact gene expression. Interestingly, variability in the promoter region of virulence factor *BAD1* has been documented between the species (Meece *et al.* 2010; Kaplan *et al.* 2021). In addition, many SNV differences exist across the genomes of *B. dermatitidis* and *B. gilchristii.* Likewise, nonsynonymous SNV differences in coding regions may impact gene function, which could also lead to subtle phenotypic differences between the species.

TEs are ubiquitous in fungal genomes although their genomic proportion varies widely (Muszewska *et al.* 2011), typically ranging from 0.02–30% (Castanera *et al.* 2016) but can be as high as 74% (Frantzeskakis *et al.* 2018). *Blastomyces* spp. are unique in that a large proportion (∼60%) of their genomes are comprised of TEs. Similar to other fungi, the vast majority of TEs of both species examined in this study consisted of class I LTR-RT elements, particularly Gypsy elements (Muszewska *et al.* 2011; Grandaubert *et al.* 2014; Castanera *et al.* 2016; Kirkland *et al.* 2018; Rao *et al.* 2018; Oggenfuss *et al.* 2021). Most elements were remnant fragments with few complete LTR-RTs detected. Degraded TE remnants are common in fungal genomes (Muszewska *et al.* 2019). The genome size difference between *B. dermatitidis* and *B. gilchristii* was attributed almost completely to the presence of excess LTR-RTs in the *B. gilchristii* genome. The 2 species possessed the same LTR-RT elements but the quantities of certain elements contained in their respective genomes differ; some elements were more abundant in *B. dermatitidis* while others were more abundant in *B. gilchristii.*

Several studies describe genomic TE invasions in fungi which increased genome size and impacted evolution and speciation (Grandaubert *et al.* 2014; Castanera *et al.* 2016; Faino *et al.* 2016; Oggenfuss *et al.* 2021). Whereas many studies describe relatively young TEs as a results of recent transposition (Castanera *et al.* 2016; Horns *et al.* 2017; Rao *et al.* 2018; Lorrain *et al.* 2021; Oggenfuss *et al.* 2021), the TEs of *B. dermatitidis* and *B. gilchristii* described in this study were relatively ancient. We hypothesize that TE genome invasion and expansion accompanied or was possibly instrumental in the evolution of a *B. dermatitidis/gilchristii* common ancestor, differentiating it from other members of the genus and from other Ajellomycetaceae and Onygenales. This is supported by the observation that, despite approximately the same number of genes (Muñoz *et al.* 2015), the genomes of *B. dermatitidis* and *B. gilchristii* are more than twice the size of other closely related species: *B. parvus* (27.8Mb), *B. percursus* (32.3Mb) (Dukik *et al.* 2017), *B. silverae* (30.4 Mb), *Emmonsia crescens* (30.7 Mb) (Muñoz *et al.* 2015), *B. emzantsi* (34.05 Mb) (Schwartz *et al.* 2021), *H. capsulatum* (32.5 Mb) (Voorhies *et al.* 2022), *Paracoccidioides brasiliensis* (30.0 Mb), *Paracoccidioides lutzii* (32.9 Mb) (Desjardins *et al.* 2011), *Coccidioides immitis* (28.9 Mb), and *Coccidioides posadasii* (27 Mb) (Neafsey *et al.* 2010).

Notwithstanding the genome size difference between *B. dermatitidis* and *B. gilchristii* primarily attributed to Gypsy LTR-RT elements, several lines of evidence suggest that the insertion of these TEs predates the species’ divergence, negating LTR-RT transpositional proliferation as the cause of the genome size difference between the species. The LTR-RT families are highly divergent suggesting that they are ancient, predating the speciation of *B. dermatitidis* and *B. gilchristii*, estimated at 1.9 MYA (95% HPD 0.5–4.4 MYA) (McTaggart *et al.* 2016). The ancient age of *Blastomyces* LTR-RTs was estimated by 2 methods: (1) divergence from the repeat family consensus sequence applied to all elements and (2) divergence of the 3′ and 5′ LTR regions of intact LTR-RTs only, with similar results. Both dating methods are commonly applied to LTR-RTs for age estimation, although shortcomings are noted for both methods (Maumus and Quesneville 2014; Kapusta and Suh 2017; Jedlicka *et al.* 2020). The age estimates for the LTR-RTs are dependent on both the sequence divergence and the substitution rate parameter and binned in 5 MYA increments to account for error. Consequently, the age estimates should be interpreted with caution because LTR-RTs are likely subject to higher mutation rates than the neutrally evolving sequences since error-prone transcription and reverse transcription precede new insertions. Likewise, LTR-RTs are susceptible to repeat-induced point mutation, which would elevate the mutation rate. Hence, the age of these TEs may be overestimated.

Nevertheless, additional observations support the hypothesis that the proliferation of Gypsy LTR-RTs predated the speciation of *B. dermatitidis* and *B. gilchristii*. Few of the LTR-RT elements in are ER-3 and SLH14081 are intact and none contain all of the functional domains for transposition, suggesting that none are currently active. Incomplete and nonfunctional LTR-RTs are common in fungi (Muszewska *et al.* 2011). Furthermore, TE content in *B. dermatitidis* and *B. gilchristii* is compartmentalized into large isochore regions of high and low GC content (Muñoz *et al.* 2015). This suggests a period of time since insertion for chromosomal rearrangements and purifying selection of deleterious TE insertions in exonic sequences to establish TE compartmentalization, and it contrasts with the lack of compartmentalization observed in genomes such as *Blumeria graminis* with a recent TE expansion (Frantzeskakis *et al.* 2018; Oggenfuss *et al.* 2021). Finally, phylogenetic analysis of reverse transcriptase domains of 3 Gypsy LTR-RT elements and the Copia LTR-RT elements shows no strong phylogenetic distinction between species. The analysis suggests that transposition occurred before species divergence, followed by independent evolution of the domains in each of the species.

Genome size is determined by the opposing forces of genome expansion often mediated by TE transposition and genome contraction that occurs when sequences are excised by homologous ectopic recombination between repeat copies or nonspecific DNA loss through double-strand break repair (Devos *et al.* 2002; Macas *et al.* 2015; Faino *et al.* 2016). We postulate that the difference in genome size and LTR-RT content between the *B. dermatitidis* and *B. gilchristii* genomes is due to different evolutionary rates and trajectories of genome contraction. This is consistent with the observation that the species contain differing amounts of the same LTR-RT elements. Although genome contraction is difficult to detect through whole genome sequence analysis, the abundance of solo-LTRs in genomes of both species is evidence for extensive genome contraction because solo-LTRs are the byproduct of homologous recombination of the 3′ and 5′ LTR regions either within or between repeat copies leading to the excision of the internal sequence and 1 of the LTR regions (Macas *et al.* 2015). Different rates of genome contraction may also explain why the LTR-RT elements appeared more divergent and older in *B. gilchristii*. In a study of TEs in different bird lineages, flightless birds had larger genomes and older TEs compared to flying birds due to relatively slower removal of DNA from their genomes (Kapusta *et al.* 2017). TEs are known to mediate reproductive segregation leading to speciation by creating chromosomal rearrangements through homologous recombination and alternative transposition that prevent effective meiotic recombination between lineages (Gray 2000; Grandaubert *et al.* 2014; Serrato-Capuchina and Matute 2018). The *MAT* locus in *Blastomyces* is an example of alternate genome locus structure in *B. dermatitidis* and *B. gilchristii* caused by variable TE presence (Li *et al.* 2013). Thus, we hypothesize that following TE and specifically LTR-RT expansion concurrent with the evolution of a *B. dermatitidis/gilchristii* common ancestor, different trajectories of genome contraction contributed to the evolution *B. dermatitidis* and *B. gilchristii* by facilitating and/or maintaining reproductive isolation.

Phylogeographic analysis suggests that *B. dermatitidis* is present throughout the eastern half of North America whereas *B. gilchristii* is restricted to select Canadian provinces and some northern US states (McTaggart *et al.* 2016). With species divergence estimated at 1.9 MYA (95% HPD 0.5–4.4 MYA) during the Pleistocene epoch, it has been postulated that North America glaciations during this period created biotic refugia, providing an impetus for speciation (McTaggart *et al.* 2016). Under this scenario, repeated glaciation would have caused geographic isolation of subsets of populations creating the opportunity for alternate trajectories of genome contraction leading to speciation. The more southerly *B. dermatitidis* ancestors may have had more opportunity for recombination necessary for genome contraction (Devos *et al.* 2002) while the northern *B. gilchristii* ancestors remained frozen in ice for thousands of years. This may explain why the *B. dermatitidis* genomes are smaller than those of *B. gilchristii*.

In conclusion, we characterize genomic differences between cryptic species *B. dermatitidis* and *B. gilchristii*, the causative agents of blastomycosis. Future research involving additional and more complete genome sequences will be invaluable in understanding the genetic traits that correlate with the clinical outcomes and ecological variations of these 2 important pathogens. *De novo* sequencing and analysis of multiple isolates of each species confirms that ancient TE content, in particular Gypsy LTR-RT content, is a major genomic difference between *B. dermatitidis* and *B. gilchristii*. Differential TE genomic components likely reinforce reproductive isolation of the 2 species and highlight the importance of the “repeatome” in fungal speciation. However, TEs are also postulated to modulate gene expression usually through TE-mediated transcriptional repression or silencing (Castanera *et al.* 2016; Torres *et al.* 2021). Future studies on genes proximal to TEs represent a promising avenue of research to further elucidate the full impact of the differential TE content on species-specific adaptations, fungal lifestyle traits, and virulence.

## Supplementary Material

jkae194_Supplementary_Data

## Data Availability

Sequencing raw reads, de novo assemble genomes and genome annotations are available in the NCBI Sequence Read Archive and GenBank under Bioproject number PRJNA890593. Accession numbers are listed in Supplementary Table 1. Supplemental material available at G3 online.
