# Supplementary material for: Comparative genome analysis and the genome-shaping role of long terminal repeat retrotransposons in the evolutionary divergence of fungal pathogens Blastomyces dermatitidis and Blastomyces gilchristii
Source: G3 (Bethesda). 2024 Aug 20;14(11):jkae194. doi: 10.1093/g3journal/jkae194 (PMC11540331; doi:10.1093/g3journal/jkae194)
Supplement: jkae194_Supplementary_Data [file jkae194_supplementary_data.zip › Supplemental_Figure_Legends_G3-2024-405110.docx]

Figure S1: Frequency distribution of percentage of GC content of short read sequences.

Figure S2: Syntenic comparison of the *MAT* locus of *B. dermatitidis* and *B. gilchristii* isolates. The *MAT* locus is bounded by *SlaB*, cytochrome c oxidase subunit 6a (*COX13)* and AP endonuclease-2 (*APN2)* genes. Red bars represent highly syntenic and similar DNA regions, while dark blue bars represent highly syntenic and similar DNA regions in the reverse alignment. Isolates designated as mating type (+) contain the *MAT1-1* locus with the alpha-box gene, while those designated as mating type (-) contain the *MAT1-2* locus with the HMG domain. TE sequence is designated in light blue.
