# Supplementary material for: Comparative genome analysis and the genome-shaping role of long terminal repeat retrotransposons in the evolutionary divergence of fungal pathogens Blastomyces dermatitidis and Blastomyces gilchristii
Source: G3 (Bethesda). 2024 Aug 20;14(11):jkae194. doi: 10.1093/g3journal/jkae194 (PMC11540331; doi:10.1093/g3journal/jkae194)
Supplement: jkae194_Supplementary_Data [file jkae194_supplementary_data.zip › Supplemental_Material_-_Table_S1_and_Table_S2_G3-2024-405110.docx]

Table S1: List of study samples

| Species | Sample No. | Location | SRA No. | GenBank Accession No. | Reference |
| --- | --- | --- | --- | --- | --- |
| *B. dermatitidis* | 22281 | Ontario, Canada | SRR25928116 | JAVKUR000000000 | This study |
|  | 23166 | Ontario, Canada | SRR25928124 | JAVKUP000000000 | This study |
|  | 19X135 | Ontario, Canada | SRR21903595 |  | This study |
|  | 19X655 | Ontario, Canada | SRR21903594 |  | This study |
|  | 19X404 | Ontario, Canada | SRR21903583 |  | This study |
|  | 19X544 | Ontario, Canada | SRR21903572 |  | This study |
|  | 19X233 | Ontario, Canada | SRR21903571 |  | This study |
|  | 20X751 | Ontario, Canada | SRR21903570 |  | This study |
|  | 20X534 | Ontario, Canada | SRR21903569 |  | This study |
|  | 20X787 | Ontario, Canada | SRR21903568 |  | This study |
|  | 21X398 | Ontario, Canada | SRR21903567 |  | This study |
|  | 21X689 | Ontario, Canada | SRR25928115 |  | This study |
|  | 21X936 | Ontario, Canada | SRR25928114 |  | This study |
|  | 22X682 | Ontario, Canada | SRR25928123 |  | This study |
|  | 22X8264 | Ontario, Canada | SRR25928122 |  | This study |
|  | 22X840 | Ontario, Canada | SRR25928121 |  | This study |
|  | 21X714 | Ontario, Canada | SRR21903593 |  | This study |
|  | 21X297 | Ontario, Canada | SRR21903592 |  | This study |
|  | MYCO-00930 | Quebec, Canada | SRR15390213 |  | (Carignan *et al.* 2021) |
|  | MYCO-01443 | Quebec, Canada | SRR15390231 |  | (Carignan *et al.* 2021) |
|  | LSPQ-00853 | Quebec, Canada | SRR15390241 |  | (Carignan *et al.* 2021) |
|  | LSPQ-00867 | Quebec, Canada | SRR15390245 |  | (Carignan *et al.* 2021) |
|  | LSPQ-00918 | Quebec, Canada | SRR15390249 |  | (Carignan *et al.* 2021) |
|  | LSPQ-00973 | Quebec, Canada | SRR15390252 |  | (Carignan *et al.* 2021) |
|  | LSPQ-01013 | Quebec, Canada | SRR15390257 |  | (Carignan *et al.* 2021) |
|  | LSPQ-01102 | Quebec, Canada | SRR15390264 |  | (Carignan *et al.* 2021) |
|  | LSPQ-01254 | Quebec, Canada | SRR15390284 |  | (Carignan *et al.* 2021) |
|  | LSPQ-01266 | Quebec, Canada | SRR15390288 |  | (Carignan *et al.* 2021) |
|  | LSPQ-00713 | Quebec, Canada | SRR15390305 |  | (Carignan *et al.* 2021) |
|  | LSPQ-00940 | Quebec, Canada | SRR15390313 |  | (Carignan *et al.* 2021) |
|  | LSPQ-00976 | Quebec, Canada | SRR15390314 |  | (Carignan *et al.* 2021) |
|  | LSPQ-01003 | Quebec, Canada | SRR15390316 |  | (Carignan *et al.* 2021) |
|  | LSPQ-00672 | Quebec, Canada | SRR15390317 |  | (Carignan *et al.* 2021) |
|  | LSPQ-01016 | Quebec, Canada | SRR15390319 |  | (Carignan *et al.* 2021) |
|  | LSPQ-00499 | Quebec, Canada | SRR15390326 |  | (Carignan *et al.* 2021) |
|  | MYCO-00895 | Quebec, Canada | SRR15390334 |  | (Carignan *et al.* 2021) |
|  | MYCO-00859 | Quebec, Canada | SRR15390351 |  | (Carignan *et al.* 2021) |
|  | ER3 |  | SRR11849827 | GCA_000003525.2 | (Muñoz *et al.* 2015) |
|  | ATCC26199 |  | SRR11849828 |  | (Muñoz *et al.* 2015) |
|  | ATCC18188 |  | SRR11849829 |  | (Muñoz *et al.* 2015) |
| *B. gilchristii* | 22264 | Ontario, Canada | SRR25928117 | JAVKUS000000000 | This study |
|  | 23019 | Ontario, Canada | SRR25928113 | JAVKUQ000000000 | This study |
|  | 19X611 | Ontario, Canada | SRR21903591 |  | (McTaggart *et al.* 2024) |
|  | 19X159 | Ontario, Canada | SRR21903581 |  | (McTaggart *et al.* 2024) |
|  | 19X301 | Ontario, Canada | SRR21903590 |  | (McTaggart *et al.* 2024) |
|  | 19X280 | Ontario, Canada | SRR21903589 |  | (McTaggart *et al.* 2024) |
|  | 19X542 | Ontario, Canada | SRR21903588 |  | (McTaggart *et al.* 2024) |
|  | 20X504 | Ontario, Canada | SRR21903587 |  | (McTaggart *et al.* 2024) |
|  | 20X036 | Ontario, Canada | SRR21903586 |  | (McTaggart *et al.* 2024) |
|  | 20X822 | Ontario, Canada | SRR21903585 |  | (McTaggart *et al.* 2024) |
|  | 20X548 | Ontario, Canada | SRR21903584 |  | (McTaggart *et al.* 2024) |
|  | 20X814 | Ontario, Canada | SRR21903582 |  | (McTaggart *et al.* 2024) |
|  | 21X982 | Ontario, Canada | SRR21903579 |  | (McTaggart *et al.* 2024) |
|  | 21X597 | Ontario, Canada | SRR21903578 |  | (McTaggart *et al.* 2024) |
|  | 21X285 | Ontario, Canada | SRR21903577 |  | (McTaggart *et al.* 2024) |
|  | 21X378 | Ontario, Canada | SRR21903576 |  | (McTaggart *et al.* 2024) |
|  | 21X805 | Ontario, Canada | SRR21903575 |  | (McTaggart *et al.* 2024) |
|  | 22X325 | Ontario, Canada | SRR25928120 |  | (McTaggart *et al.* 2024) |
|  | 22X343 | Ontario, Canada | SRR25928119 |  | (McTaggart *et al.* 2024) |
|  | 22X864 | Ontario, Canada | SRR25928118 |  | (McTaggart *et al.* 2024) |
|  | 19X156 | Ontario, Canada | SRR21903574 |  | (McTaggart *et al.* 2024) |
|  | 21X289 | Ontario, Canada | SRR21903573 |  | (McTaggart *et al.* 2024) |
|  | MYCO-00852 | Quebec, Canada | SRR15390308 |  | (Carignan *et al.* 2021) |
|  | LSPQ-00985 | Quebec, Canada | SRR15390315 |  | (Carignan *et al.* 2021) |
|  | LSPQ-00666 | Quebec, Canada | SRR15390327 |  | (Carignan *et al.* 2021) |
|  | B19407 | Minnesota, USA | SRR17219283 |  | (Bagal *et al.* 2022) |
|  | B19406 | Minnesota, USA | SRR17219284 |  | (Bagal *et al.* 2022) |
|  | SLH14081 |  |  | GCF_000003855.2 | (Muñoz *et al.* 2015) |
| *B. emzantsi* | MRL_BACN2 | South Africa |  | GCA_003206195.1 | (Maphanga *et al.* 2020) |
|  | MRL_BACN5 | South Africa |  | GCA_003206205.1 | (Maphanga *et al.* 2020) |
|  | MRL_BACNE1992 | South Africa |  | GCA_003206725.1 | (Maphanga *et al.* 2020) |
|  | MRL_BADD | South Africa |  | GCA_003206745.1 | (Maphanga *et al.* 2020) |
|  | MRL_BASAMIR | South Africa |  | GCA_003206755.1 | (Maphanga *et al.* 2020) |
|  | MRL_BACNC | South Africa |  | GCA_003206845.1 | (Maphanga *et al.* 2020) |
|  | MRL_BACN3 | South Africa |  | GCA_003226315.1 | (Maphanga *et al.* 2020) |
| *B. parvus* | UAMH130 | Arizona, USA |  | GCA_002572885.1 | (Muñoz *et al.* 2015; Jiang *et al.* 2018) |
| *B. percursus* | IHEM26956 | RSA |  | GCA_018296065.1 | (Schwartz *et al.* 2021) |
|  | IHEM26957 | Morocco |  | GCA_018296075.1 | (Schwartz *et al.* 2021) |
|  | IHEM26955 | DRC |  | GCA_018296045.1 | (Schwartz *et al.* 2021) |
|  | IHEM26951 | Uganda |  | GCA_018296055.1 | (Schwartz *et al.* 2021) |
|  | EI222 | South Africa |  | GCA_001883805.1 | (Maphanga *et al.* 2020) |
|  | MRL_BPX888 | South Africa |  | GCA_003206225.1 | (Maphanga *et al.* 2020) |
|  | MRL_BPCN13 | South Africa |  | GCA_003206275.1 | (Maphanga *et al.* 2020) |
|  | MRL_BPOM | South Africa |  | GCA_003206295.1 | (Maphanga *et al.* 2020) |
|  | MRL_BP13 | South Africa |  | GCA_003206765.1 | (Maphanga *et al.* 2020) |
|  | MRL_BP1777 | South Africa |  | GCA_003206785.1 | (Maphanga *et al.* 2020) |
|  | MRL_BPSD | South Africa |  | GCA_003206805.1 | (Maphanga *et al.* 2020) |
|  | MRL_BPM124 | South Africa |  | GCA_003206875.1 | (Maphanga *et al.* 2020) |
|  | MRL_BPZV | South Africa |  | GCA_003206885.1 | (Maphanga *et al.* 2020) |
|  | MRL_BPNCPF4091 | South Africa |  | GCA_003417775.1 | (Maphanga *et al.* 2020) |
| *B. silverae* | UAMH139 | Montana, USA |  | GCA_001014755.1 | (Muñoz *et al.* 2015; Jiang *et al.* 2018) |

Table S2: Percent average nucleotide identity generated by OrthoANI for *de* *novo* assembled genomes of *B. dermatitidis* and *B. gilchristii*

|  |  | *B. dermatitidis* | | | *B. gilchristii* | | |
| --- | --- | --- | --- | --- | --- | --- | --- |
|  |  | ER3 | 22281 | 23166 | 22264 | 23019 | SLH14081 |
| *B. dermatitidis* | ER3 |  |  |  |  |  |  |
|  | 22281 | 97.25 |  |  |  |  |  |
|  | 23166 | 97.12 | 99.47 |  |  |  |  |
| *B. gilchristii* | 22264 | 96.60 | 96.58 | 96.57 |  |  |  |
|  | 23019 | 96.60 | 96.57 | 96.59 | 99.44 |  |  |
|  | SLH14081 | 96.60 | 96.57 | 96.53 | 98.75 | 98.72 |  |

**REFERENCES**

Bagal UR, Ireland M, Gross A, Fischer J, Bentz M, *et al.* 2022. Molecular Epidemiology of Blastomyces gilchristii Clusters, Minnesota, USA. Emerg. Infect. Dis. 28 (9): 1924–1926. doi: 10.3201/eid2809.220392.

Carignan A, Boudhrioua C, Moreira S, Pelletier AA, Dufour K, *et al.* 2021. Changing patterns of disease severity in Blastomyces dermatitidis infection, Quebec, Canada. Emerg. Infect. Dis. 27 (11): 2810–2816. doi: 10.3201/eid2711.210552.

Jiang Y, Dukik K, Muñoz JF, Sigler L, Schwartz IS, *et al.* 2018. Phylogeny, ecology and taxonomy of systemic pathogens and their relatives in Ajellomycetaceae (Onygenales): Blastomyces, Emergomyces, Emmonsia, Emmonsiellopsis. Fungal Divers. 90 (1): 245–291. doi: 10.1007/s13225-018-0403-y.

Maphanga TG, Birkhead M, Muñoz JF, Allam M, Zulu TG, *et al.* 2020. Human Blastomycosis in South Africa Caused by Blastomyces percursus and Blastomyces emzantsi sp. nov., 1967 to 2014. J. Clin. Microbiol. 58 (3): e01661-19. doi: 10.1128/JCM.01661-19.

McTaggart LR, Varghese N, Sivaraman K, Patel SN, Kus J V. 2024. Genomic investigation of a blastomycosis outbreak. Emerg. Infect. Dis. in press.

Muñoz JF, Gauthier GM, Desjardins CA, Gallo JE, Holder J, *et al.* 2015. The Dynamic Genome and Transcriptome of the Human Fungal Pathogen Blastomyces and Close Relative Emmonsia. PLoS Genet. 11 (10): e1005493. doi: 10.1371/journal.pgen.1005493.

Schwartz IS, Muñoz JF, Kenyon CR, Govender NP, McTaggart L, *et al.* 2021. Blastomycosis in Africa and the Middle East: A Comprehensive Review of Reported Cases and Reanalysis of Historical Isolates Based on Molecular Data. Clin. Infect. Dis. 73 (7): e1560–e1569. doi: 10.1093/cid/ciaa1100.
