# Supplementary figures and images for: Comparative genome analysis and the genome-shaping role of long terminal repeat retrotransposons in the evolutionary divergence of fungal pathogens Blastomyces dermatitidis and Blastomyces gilchristii
Source: G3 (Bethesda). 2024 Aug 20;14(11):jkae194. doi: 10.1093/g3journal/jkae194 (PMC11540331; doi:10.1093/g3journal/jkae194)

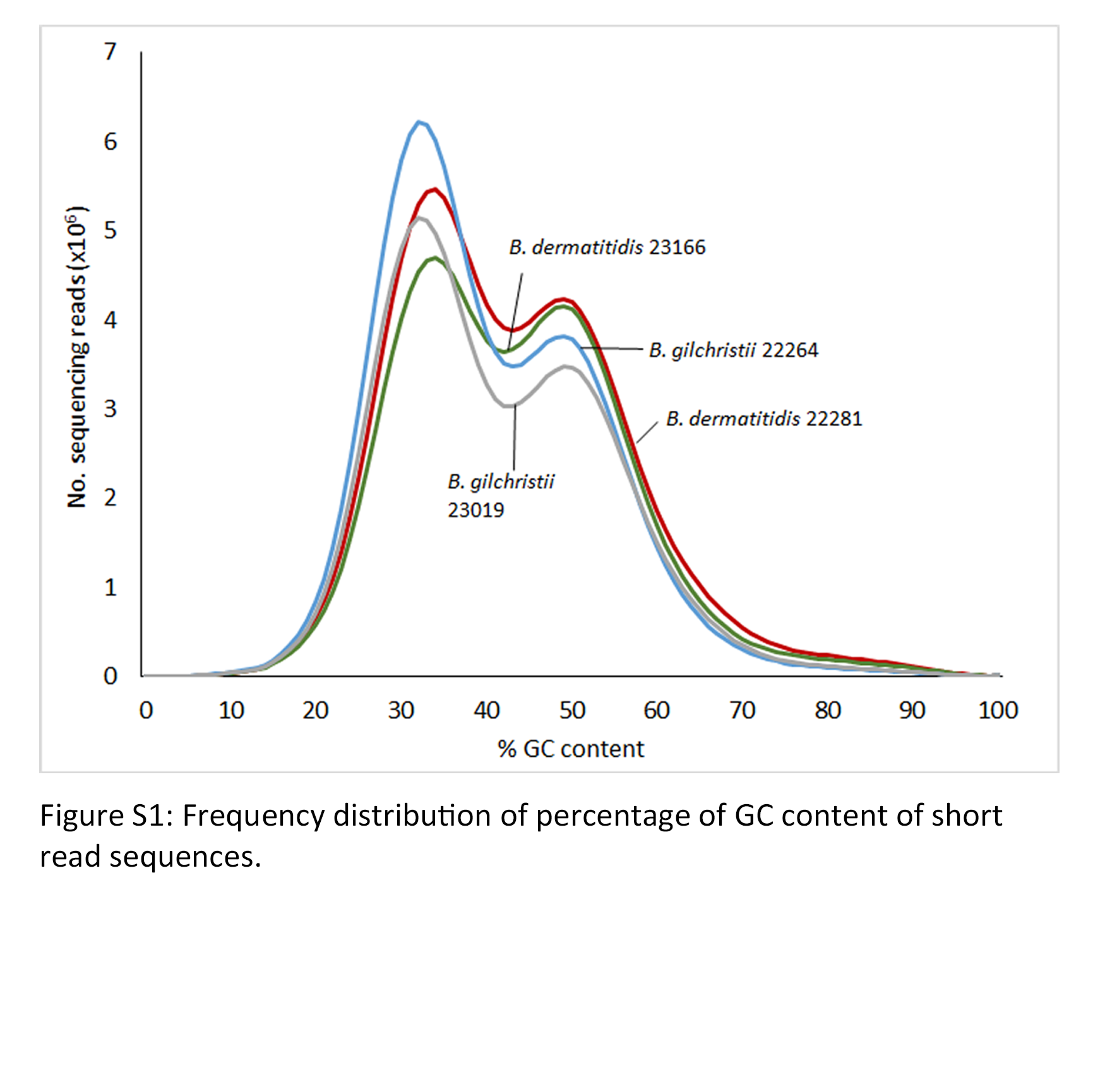

Supplement: jkae194_Supplementary_Data [file jkae194_supplementary_data.zip › Figure S1 with title.tif]

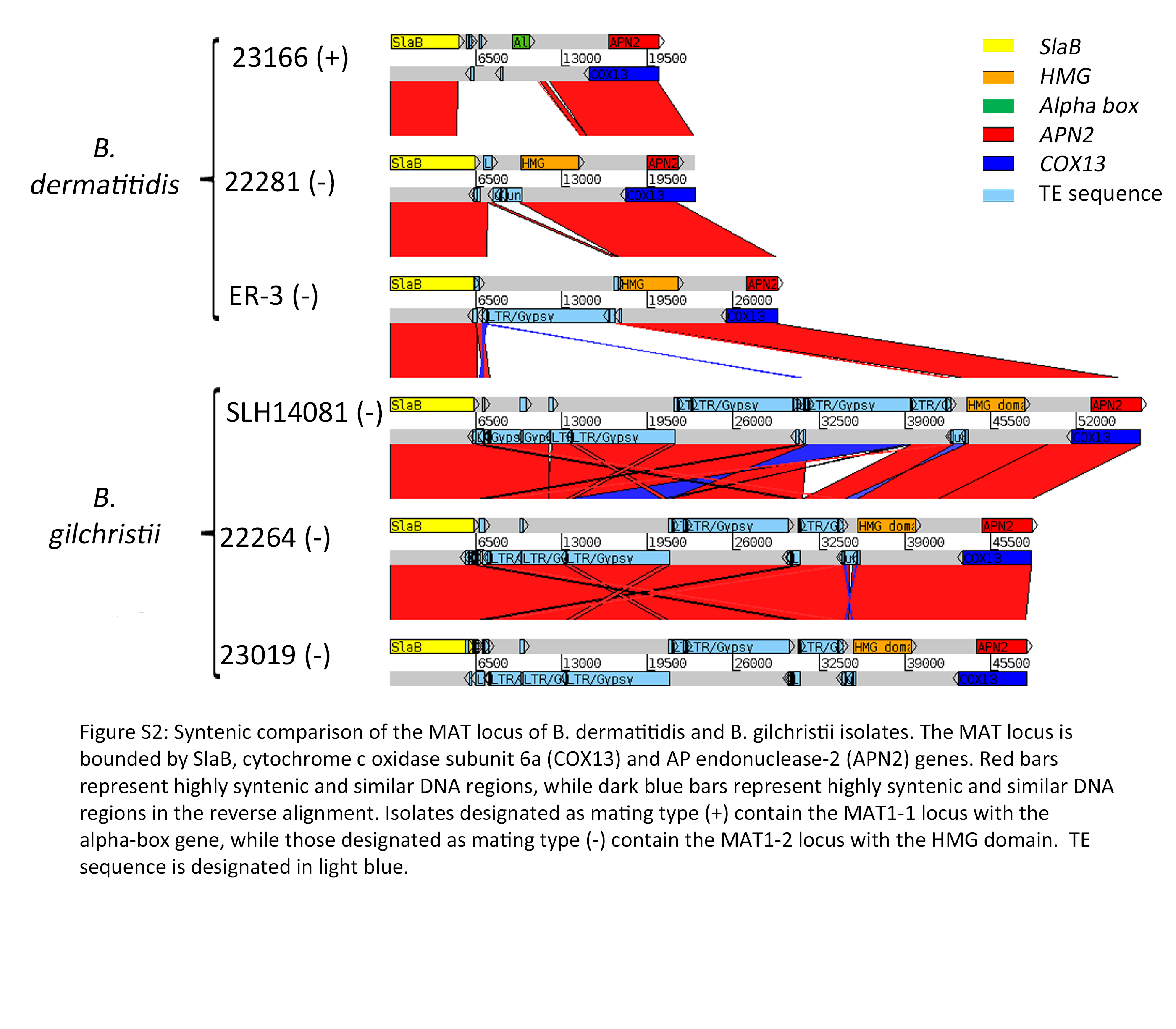

Supplement: jkae194_Supplementary_Data [file jkae194_supplementary_data.zip › Figure S2 with title.tif]
